# Loss of NF1 Expression in Human Endothelial Cells Promotes Autonomous Proliferation and Altered Vascular Morphogenesis

**DOI:** 10.1371/journal.pone.0049222

**Published:** 2012-11-07

**Authors:** Anshika Bajaj, Qing-fen Li, Qingxia Zheng, Kevin Pumiglia

**Affiliations:** Center for Cell Biology and Cancer Research, Albany Medical College, Albany, New York, United States of America; Duke University Medical Center, United States of America

## Abstract

Neurofibromatosis is a well known familial tumor syndrome, however these patients also suffer from a number of vascular anomalies. The loss of NFl from the endothelium is embryonically lethal in mouse developmental models, however little is known regarding the molecular regulation by NF1 in endothelium. We investigated the consequences of losing NF1 expression on the function of endothelial cells using shRNA. The loss of NF1 was sufficient to elevate levels of active Ras under non-stimulated conditions. These elevations in Ras activity were associated with activation of downstream signaling including activation of ERK, AKT and mTOR. Cells knocked down in NF1 expression exhibited no cellular senescence. Rather, they demonstrated augmented proliferation and autonomous entry into the cell cycle. These proliferative changes were accompanied by enhanced expression of cyclin D, phosphorylation of p27^KIP^, and decreases in total p27^KIP^ levels, even under growth factor free conditions. In addition, NF1-deficient cells failed to undergo normal branching morphogenesis in a co-culture assay, instead forming planar islands with few tubules and branches. We find the changes induced by the loss of NF1 could be mitigated by co-expression of the GAP-related domain of NF1 implicating Ras regulation in these effects. Using doxycycline-inducible shRNA, targeting NF1, we find that the morphogenic changes are reversible. Similarly, in fully differentiated and stable vascular-like structures, the silencing of NF1 results in the appearance of abnormal vascular structures. Finally, the proliferative changes and the abnormal vascular morphogenesis are normalized by low-dose rapamycin treatment. These data provide a detailed analysis of the molecular and functional consequences of NF1 loss in human endothelial cells. These insights may provide new approaches to therapeutically addressing vascular abnormalities in these patients while underscoring a critical role for normal Ras regulation in maintaining the health and function of the vasculature.

## Introduction

Mutations in the NF1 gene cause Neurofibromatosis type 1, an autosomal dominant disease that affects approximately 1 in 3000 individuals, making it one of the most common inherited genetic disorders [1]. NF1 has variable clinical manifestations. Most commonly observed changes include alterations in skin pigmentation (café au-lait spots as well as freckling) and the presence of benign and malignant nerve sheath tumors termed neurofibromas [1]. Importantly, a significant clinical manifestation of NF1 disease includes vascular disease. Patients with NF1 disease make up a significant portion of all those patients presenting with renal artery stenosis and early-onset cerebral vascular disease [2] and cardiovascular disease is a significant contributor to premature death in NF1 patients, particularly among younger patients. One study suggested that vasculopathy was over seven times more likely to occur in NF1 patients under 30 compared to their unaffected peers [3].

NF1 is clinically associated with a pleiotropic array of vascular abnormalities including stenosis, malformations, aneurysms, and hypertension. As a consequence these patients show a markedly elevated risk of cerebrovascular accidents [2]. Previous studies in mice have suggested an important role of smooth muscle cells [4] and bone marrow cells [5] in neointimal hyperplasia, inflammation and exaggerated response to injury including enhanced angiogenesis. However little is currently known about factors contributing to vascular malformations and the role of endothelial cells in regulating these changes. In addition, the endothelium is critically poised to regulate blood vessel formation, vascular tone, inflammation, as well as coagulation, thus a better understanding of the role of NF1 in regulating the function of the vascular endothelium may be critical to understanding many facets of this disease and reducing its morbidity.

Previous studies support the notion that NF1 has a critical role in the vascular endothelium. Deletion of NF1 from the vascular endothelium results in embryonic lethality [6] and NF1 haploinsufficient mice show exaggerated angiogenic responses [7]. As NF1 is a Ras-GTPase activating protein, changes in Ras activation are often associated with the loss of NF1 and data have been published suggesting that shRNA mediated knockdown of NF1 can augment growth factor mediated Ras activation and downstream signaling in endothelial cells [8]. NF1 is a large protein however, with other signaling effects, including changes in cAMP and mTOR, which can be Ras-independent [9]. We have recently published that activation of Ras in primary endothelial cells is sufficient to drive a pro-survival, pro-proliferative phenotype that disrupts normal vascular morphogenesis [10]. It is unclear if loss of NF1 is sufficient to enhance basal activation of Ras and initiate cellular responses in the absence of additional growth factor signaling. We conducted these studies to determine if the loss of NF1 is sufficient to initiate cellular signaling and alter endothelial cell function, to determine the role of Ras and other cellular signals acting downstream of NF1, and to evaluate how these changes affect the behavior of endothelial cells in a complex microenvironment.

## Results

### Knockdown of *NF1* in Primary Endothelial Cells Activates Ras and Downstream Signaling

**Figure 1:**
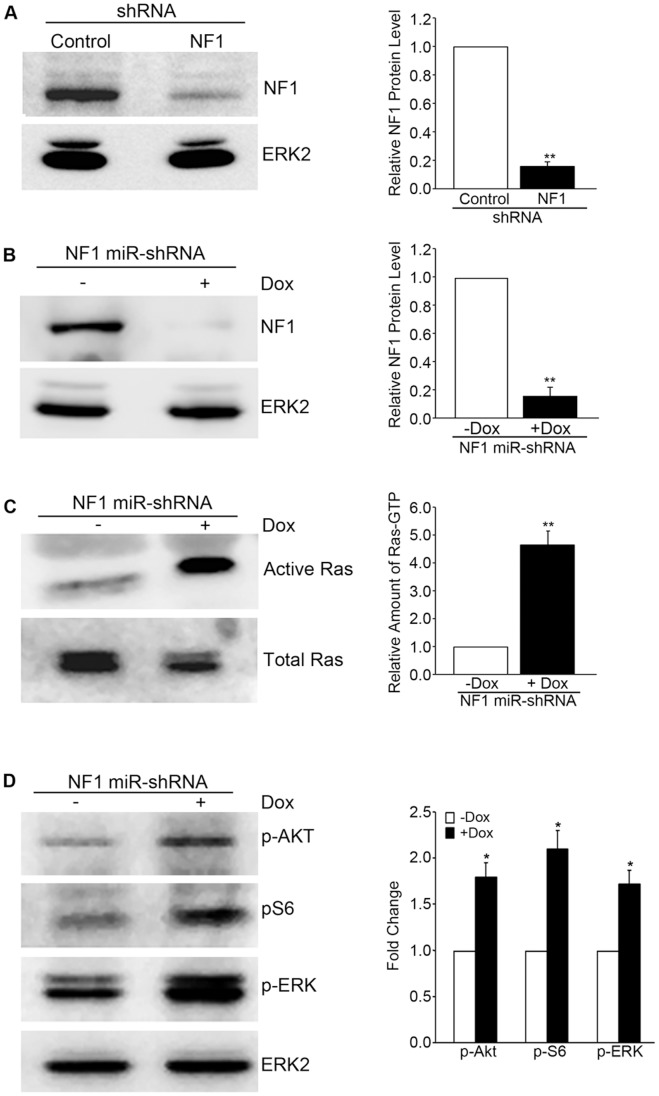
Knockdown of NF1 in Human Endothelial Cells is Sufficient to Activate Ras and Induce Cellular Signaling. (A) Early passage HUVECs were infected with a pSIREN-GFP retroviral vector carrying either a control or a NF1 shRNA and sorted for GFP expression. Western blot analysis confirmed knockdown of neurofibromin. (B) Early passage endothelial cells were infected with a pTRIPZ lentiviral vector expressing either a non-silencing control or a NF1 miR-based shRNA. The infected cells were induced with 1 µg/mL doxycycline for 48 h to induce expression of the microRNA along with red fluorescent protein (expressed in tandem) and sorted for RFP expression. Western blot confirms knockdown of neurofibromin in the presence of doxycycline while no knockdown was seen in the uninduced cells. (C) Uninduced (−Dox) and induced (+Dox) endothelial cells were serum starved for 24 h and levels of active Ras (RasGTP) were determined by using GST-Raf pull-down assay (Pierce), according to the manufacturer’s protocol. Total Ras in the total cell lysates confirmed similar amounts of protein input and was used to normalize GTP-Ras quantification. (D) Cells were treated as described above but western analysis was performed to measure activation of several key signaling proteins including phospho-Akt, phospho-S6, and phospho-ERK. Equal lysate loading was confirmed by monitoring total ERK2 levels. All experiments were performed in at least three independent sets of control and NF1 knockdown HUVECs and quantification results were averaged. (Identical results were seen with both knockdown vectors (see for example figure 6) Error bars represent standard error of the mean (**p<0.01, *p<0.05).

To generate endothelial cells lacking neurofibromin a shRNA against NF1 was cloned into pSIREN-RetroQ-ZsGreen retroviral expression vector, as previously described [8] allowing stable knockdown of neurofibromin both at the protein (Fig. 1A) and mRNA levels (not shown). Primary cells have previously demonstrated growth arrest in response to the loss of NF1 [11]. To minimize potential confounding senescence effects, to regulate expression, and also to safeguard against RNAi mediated off-target effects, a different NF1 knockdown sequence was cloned into a lentiviral pTRIPZ vector that permits inducible knockdown of the protein in the presence of 0.5 µg/mL doxycycline whereas no knockdown was seen in the absence of doxycycline (Fig. 1B). Using both types of cells we examined the levels of active Ras in quiescent cells. Knockdown of NF1 in human endothelial cells using the inducible miR-based shRNA results in increased levels of active Ras under basal conditions (Fig. 1C). Similarly, stable knockdown of NF1 also results in enhanced levels of Ras-GTP (Figure S2), even following several passages suggesting limited down-regulation or cellular compensation of this response. The enhanced levels of Ras-GTP were sufficient to trigger downstream signaling, as knockdown of NF1 resulted in enhanced activation of ERK, PI-3′-Kinase/AKT (Fig. 1D), even in the absence of any added growth factors. Another important signaling network linked to tumors and cells lacking NF1 is the mTOR signaling pathway [12], [13]. It has been shown that activation of mTOR pathway in nerve sheath tumor cell lines is essential for neurofibroma formation [14]. As the role of NF1 in regulating mTOR signaling in endothelial cells is unknown, we investigated this by examining the phosphorylation of ribosomal S6, a substrate of the TORC1 activated kinase, S6K. We found loss of NF1, even the absence of added growth factors, sufficient to stimulate mTORC1/S6 signaling. Similar effects were observed with both the inducible and the stable knockdown, suggesting little temporal or kinetic differences between the constructs or approaches (Figs. 1D and S2). These data provide evidence that knockdown of the Ras-GAP NF1 in human endothelial cells is sufficient elevate cellular GTP-Ras levels and activate downstream signaling in the absence of added growth factors.

### Cellular Proliferation in Endothelial Cells Lacking NF1

**Figure 2:**
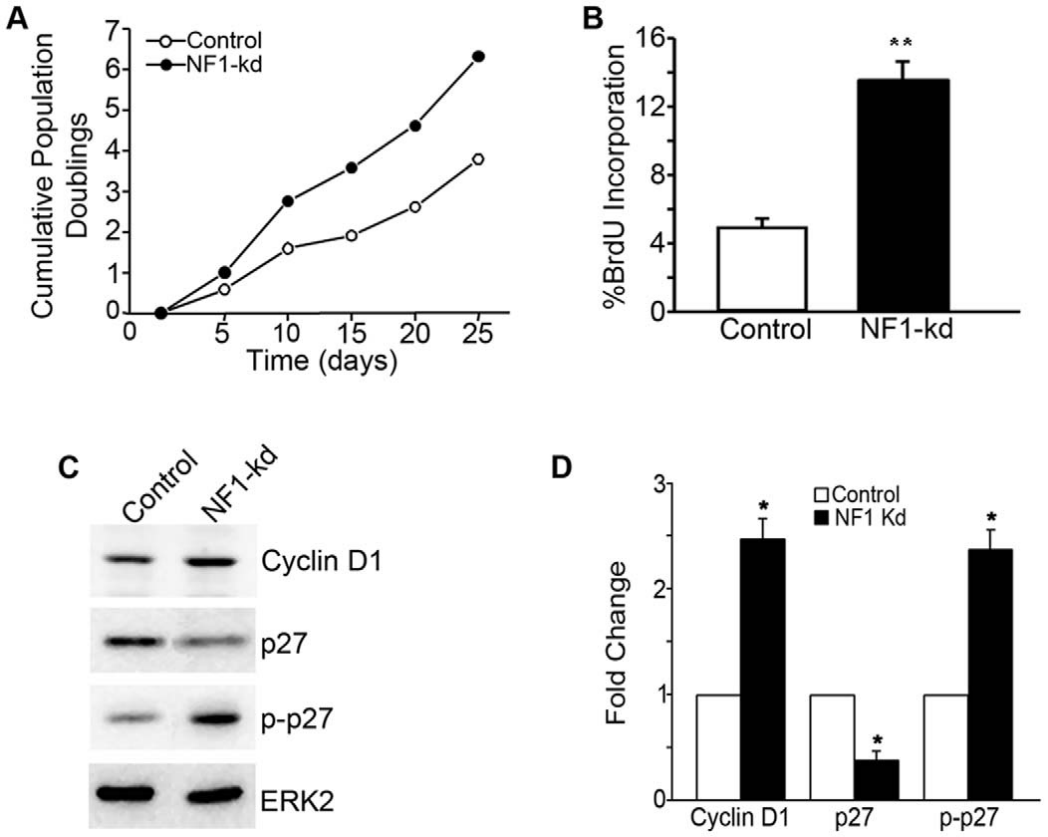
Loss of NF1 expression is sufficient to induce endothelial cell proliferation. (A) pSIREN Control and *NF1* knockdown primary endothelial cells were grown in complete media under sub-confluent conditions and cumulative population doublings were recorded over the course of 25 days as described [10]. (B) Control or NF1 knockdown endothelial cells were serum deprived for 24 h prior to cells being pulsed with BrdU for 3 h. BrdU positive cells were quantified and the data was graphed as the % positive cells compared to total cell number. Data are presented as the mean with error bars representing the standard error (**p<0.01). (C) In parallel to the experiments performed in (B), serum starved cell lysates were made after 24 h and probed with antibodies against cyclin D1, p27, phospho p27, and ERK2. ERK2 is used to insure equal loading of lysate. (D) In three independent experiments similar to those performed in (C), results were quantified and data expressed as fold-change from control. Error bars represent standard error of the mean (*p<0.05).

Down-regulation of signaling is part of a feedback response that results in replicative arrest in primary fibroblasts. However, we see no evidence of signal dampening in endothelial cells lacking NF1 even after prolonged culturing. To determine if the chronic activation of Ras and the related signaling results in replicative senescence, we examined several aspects of cellular growth. Initially, we evaluated population doublings, often used to monitor oncogene-induced senescence. In contrast to the effects observed following NF1-loss in fibroblasts [11], we found no signs of growth arrest in NF1-knockdown cells, rather they showed enhanced proliferative capacity (Fig. 2A). Given the enhanced long-term proliferative capacity of NF1-knockdown cells and the previous report that NF1-knockdown could enhance VEGF-induced proliferation, we next sought to determine if NF1 was sufficient to induce endothelial cell proliferation in the absence of growth factors. As shown in Fig. 2B, even in the absence of added mitogenic factors, endothelial cells lacking NF1 expression demonstrated enhanced entry into S-phase of the cell cycle as measured by BrdU incorporation. The enhanced proliferation was accompanied by upregulation of cyclin D levels, enhanced phosphorylation of p27KIP, and a corresponding decrease in levels of total p27 (Fig. 2C). Thus, the signaling induced by the loss of NF1 is sufficient to stimulate DNA synthesis as a consequence of cyclin D induction and the loss of the Cyclin-dependent kinase inhibitor, p27KIP.

### Loss of NF1 is Associated with Altered Vascular Morphogenesis

**Figure 3:**
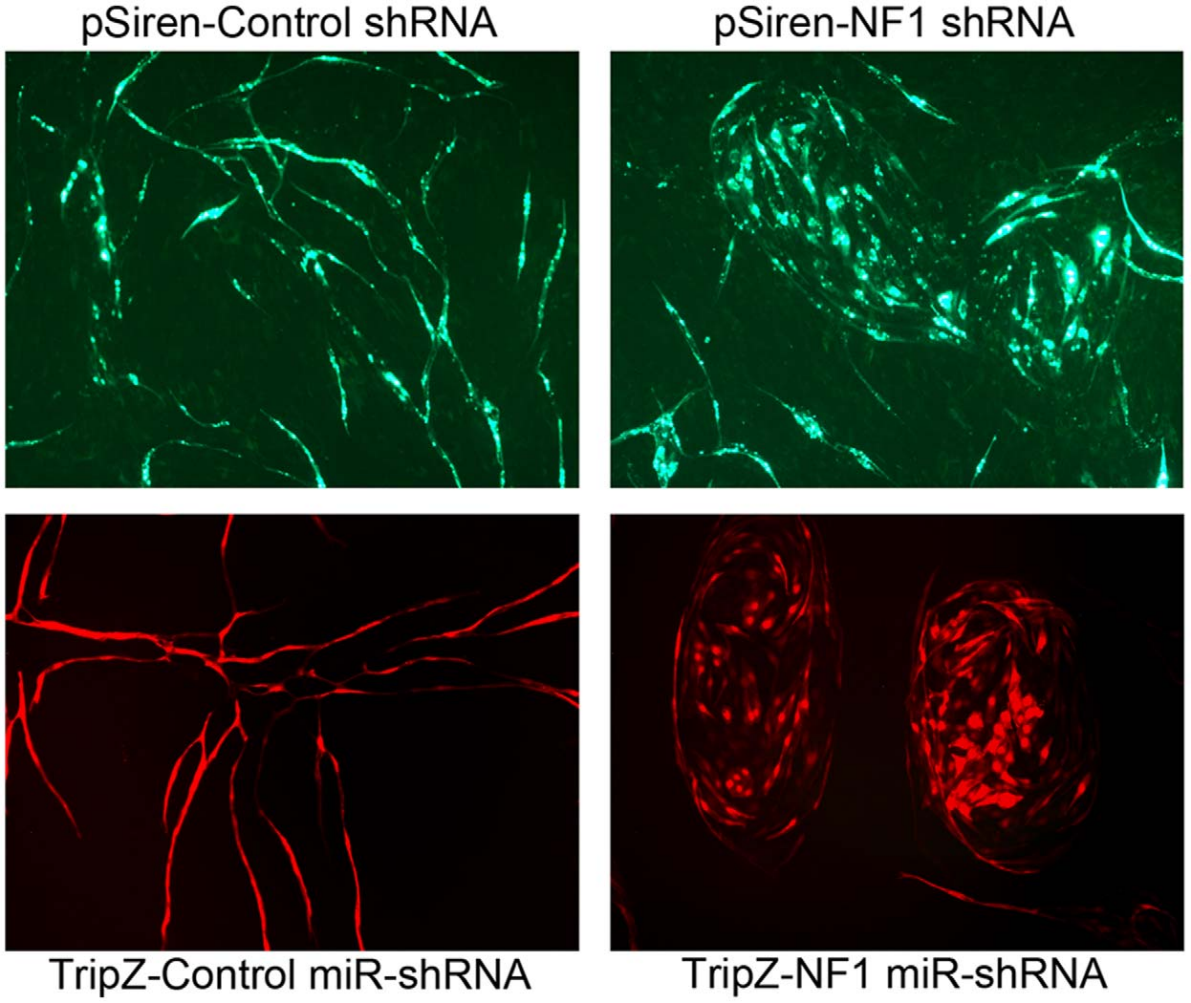
Loss of NF1 expression results in abnormal vascular morphogenesis. *Top Panel* - Primary endothelial cells infected with non-targeting pSIREN lentivirus (Control) or one directed toward knockdown of NF1 (NF1-KD). These cells were plated with primary fibroblasts in an admixed co-culture. After 14 days, endothelial cells were visualized by the vector expressed GFP. *Bottom Panel* - Endothelial cells infected with TRIPZ-Control or NF1miR-shRNA, were plated in co-culture with primary fibroblasts in the presence of doxycycline to induce expression of shRNA and RFP. Vascular structures were visualized at day 14 using RFP co-expressed in the endothelial cells.

NF1 patients are known to have several distinct types of vascular anomalies associated with the disease, including vascular malformations. At this point it is unclear if this is the result of effects arising in endothelial cells or as a consequence of an altered and pro-angiogenic microenvironment. The altered proliferative control seen upon the loss of NF1 might contribute to altered morphogenic responses by human endothelial cells. To test this we utilized a co-culture assay where human endothelial cells in the presence of human primary fibroblasts will typically proliferate for a cycle or two followed by a cessation of proliferation and the formation of branched networks of endothelial cell tubules that contain patent lumens [10], [15], [16]. When we performed this assay we found that endothelial cells lacking NF1 had an altered morphogenic response. In lieu of forming elongated and branched networks, these cells tended to form planar, sheet like structures which showed few elongations and branches, as if an essential signal to “differentiate” into vascular structures was compromised (Fig. 3). Identical phenotypes were seen whether we used constitutive shRNA (pSiren) knockdown or the inducible miR-based construct (TripZ).

### Effects on Endothelial Cells Following the Loss of NF1 are a Consequence of Ras Activation

**Figure 4:**
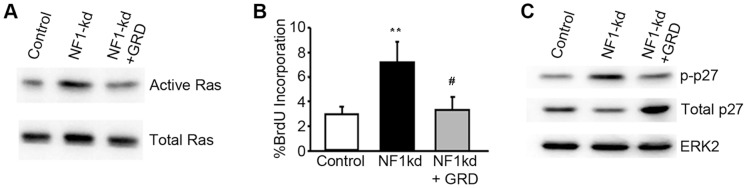
Enhanced proliferation following the loss of NF1 is a consequence of Ras activation. HUVECs were double infected with vectors coding for inducible NF1 knockdown shRNA and inducible expression of the GAP-related domain (GRD) of NF1 or empty vector. Cells were incubated for 24 h in the presence of 0.5 µg/ml doxycycline to induce expression of NF1 shRNA as well as the co-infected cDNA (GRD or empty). Control cells are cultured in the absence of doxycycline to suppress expression of and cDNA. After 24 h in the presence or absence of doxycycline, cells were switched to serum-free medium. (A) Cells were analyzed for the presence of active Ras using GST-pull down. Total Ras was used to insure equivalent protein in the input lysates. (B) BrdU incorporation was measured 24 h after changing to serum and growth factor free conditions. Data represents the averages from triplicate determinations and error bars represent standard error of the mean. (**p<0.01 compared to Control; ^#^p<0.01 compared to NF1-kd, (C) Lysates prepared under the conditions described above were probed for changes in phosphorylation of p27 and total p27 levels by western blotting. ERK was monitored as a loading control. All experiments were replicated in an independently generated set of doubly infected cells.

The loss of NF1 is sufficient to activate cellular Ras. However, NF1 is a large protein with several alternative signaling paradigms through which it can affect cell function in a Ras-independent manner, including changes in cAMP signaling [9], [17]. Therefore, we sought to determine if the effects we were seeing were the result of Ras activation. To test this we co-infected endothelial cells with inducible lentiviral vectors to knockdown NF1 and then re-express the GAP-related domain of NF1 (GRD) in order to “rescue” the Ras GAP functions of NF1 (this domain is outside the targeted region). Purified cells were obtained by two-color sorting (Red; TRIP-Z (NF1-KD) co-expressed RFP; Green; pSLIK (GRD) co-expressed YFP). As both vectors are tet-inducible, induction of knockdown also induces the “rescue” in the cells co-infected with the GRD expressing virus. As shown in Fig. 4, the expression of the GRD reverses the Ras activation seen following the loss of NF1, returning it to basal levels. This is accompanied by a similar reversal of the autonomous proliferation (Fig. 4B) that the loss of NF1 promotes. Expression of GRD also reduced the enhanced phosphorylation of p27 that is seen following the loss of NF1 with a coincident stabilization of this protein (Fig. 4C).

**Figure 5:**
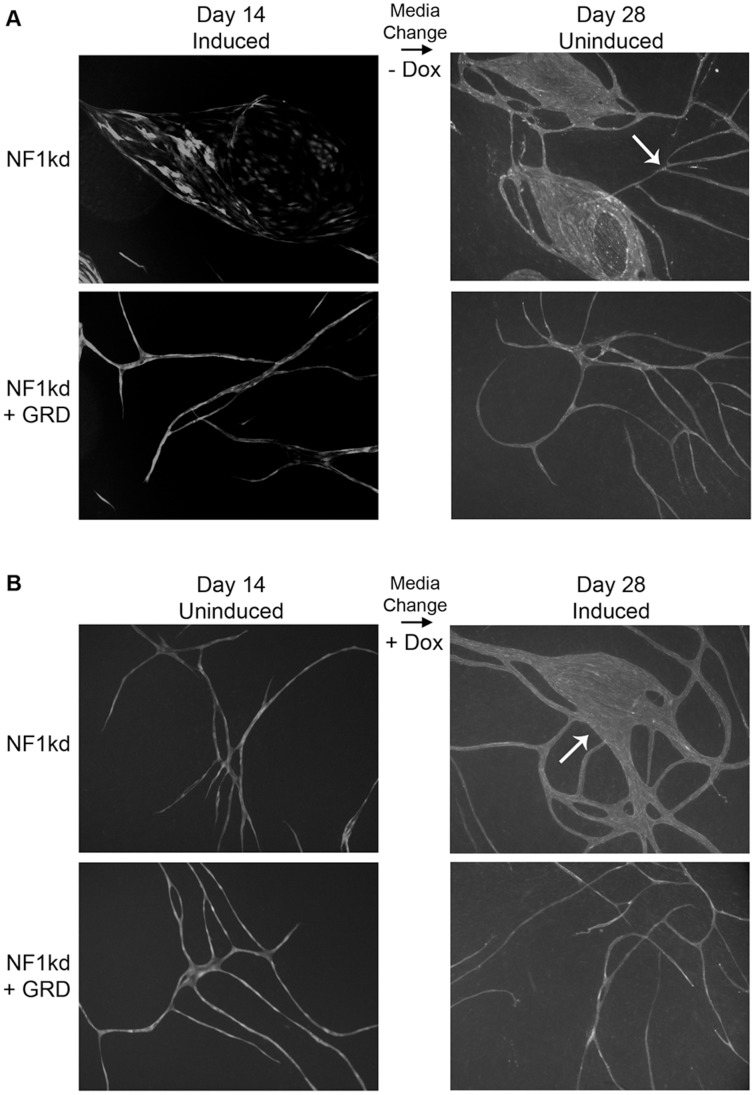
Changes in vascular morphogenesis are reversible and dependent upon active Ras. Cells for these experiments were co-infected with TRIPZ-NF1KD and pSLIK virus that was either empty or expressing the GRD domain of NF1 and sorted by FACS for double positive populations. These cells were then plated in co-culture with primary fibroblasts in the presence or absence of doxycycline as indicated. (A) Admixed cultures were allowed to form structures for 14 days. Representative fields were photographed using expressed RFP at this time and doxycycline was removed from the culture medium. Co-cultures were allowed to persist for an additional 14 days, after which time representative fields were again photographed following visualization of endothelial cells with FITC-labeled UEA-1 lectin. (B) Double positive cells were plated in the absence of doxycycline for 14 days and endothelial cells were visualized by staining live cultures with FITC labeled UEA-1 lectin and representative fields were photographed. Doxycycline was then added to cultures and they were incubated for an additional 14 days followed by visualization again using UEA-1 lectin. Arrows in both (A) and (B) highlight representative changes in morphology.

We also used these cells to determine if Ras activity was responsible for the hyperplastic morphogenic responses we observed. As shown in Fig. 5A, co-expression of the GRD reverses the abnormal morphogenesis, with cells forming nicely branched and elongating networks when the GRD domain of Ras is co-expressed. These same cells were used to explore the reversibility of this phenotype. As shown in Fig. 5A, if the NF1-kD cells are allowed to form the hyperplastic structures for 14 days (a time at which normal endothelial cell networks have stabilized) and then the Dox is removed (to cease expression of the silencing shRNA), normal looking tubular branching structures emerge over the next 14 days. We also performed the converse experiment, where normal vessel-like structures were allowed to form and stabilize over 14 days (Fig. 5B). As Dox was added to induce the knockdown of NF1, vessel-like structures begin to thicken and fuse, suggesting that malformed vascular structures can arise, even from quiescent endothelial cells. In cells “rescued” by coordinate expression of the GRD, vessel morphology remained intact and unchanged throughout the experimental manipulations. Collectively these data strongly argue that maintenance of appropriate levels of Ras activation are critical for vascular morphogenesis both in developing vessels and in established vascular networks.

### mTORC1 Activity is Essential for Endothelial Cell Proliferation and Abnormal Morphogenesis Following the Loss of NF1

**Figure 6:**
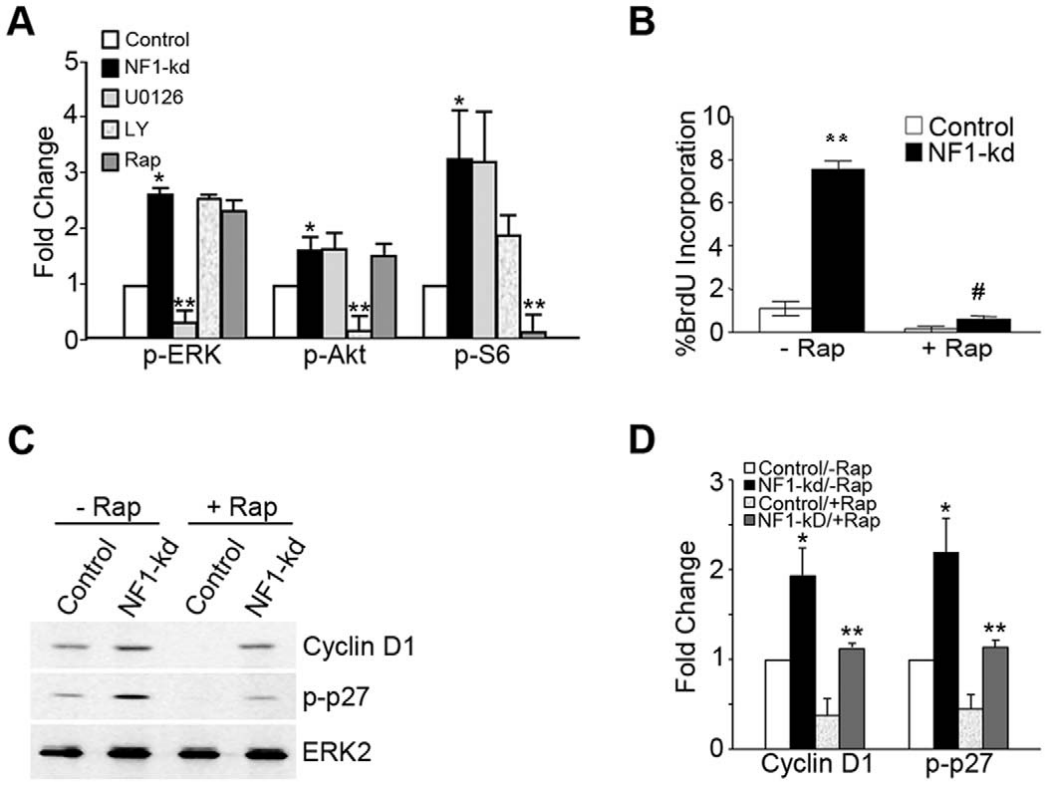
mTORC1 is critical for enhanced proliferation following the loss of NF1 (pSIREN). Control and NF1 knockdown primary endothelial cells were serum deprived for 24 h prior. Inhibitors were added was added at the time of serum starvation at the following concentrations (U0126, 1 µM; LY29002, 100 nM; Rapamycin, 0.1 ng/ml). At 21 h, some cells were pulsed with BrdU for 3 h. At 24 h cell lysates were made and probed for changes in cellular signaling (A) using phospho-specific antibodies or changes in cellular proliferation (B), visualized with an anti-BrdU antibody. (A) Results from three independent experiments were quantified from western blots similar to those shown in Supplemental 2. Data represent band average response under each condition. Error bars represent standard error of the mean and relevant statistical relationships are shown (*p<0.05 compared to Control; **p<0.01 compared to NF-kd. (B) BrdU positive cells were quantified and the data was graphed as the % positive cells compared to total cell number. The error bars represent standard error of the mean (**p<0.01 compared to control; ^#^p<0.01 compared to NF1-kd). In a similar experiment, cells was serum starved 24 h in the absence and presence of rapamycin (0.1 ng/ml) and lysates were made and probed with antibodies against cyclin D1, phospho-p27 and ERK2, the latter used as a loading control. (D) Results from three experiments similar to those performed in (C) were quantified. Data represents the mean result. Error bars represent standard error of the mean (*p<0.05 compared to control; **p<0.01 compared to NF1-kd).

We next sought to determine the mechanisms required for the autonomous proliferation seen in response to the loss of NF1 and the accompanying activation of Ras. Given the emerging role of mTOR-related signaling in other aspects of NF1 disease, we were interested in the role of this enzyme [18], [19], [20]. We used the minimal concentrations of inhibitors required to effectively inhibit Ras-related signaling back to basal levels and evaluated the effects on cellular signaling. We found we could effectively inhibit ERK activation with no significant effects on the S6 activation or AKT activation (Fig. 6A). Proliferation was completely inhibited by inhibition of MAPK, as was the induction of cyclin D1 (data not shown), consistent with previous results from our lab that have consistently found an obligatory role for MAPK signaling in the proliferative response to activated Ras and growth factors in human endothelial cells [10], [21], [22], as well as those previously reported by Munchhof et al. [8]. The effects of PI-3′-kinase inhibition were difficult to interpret, as inhibition of this signal also partially inhibited mTORC1 activation, likely explaining the intermediate and variable effect we observed with this inhibitor in proliferation assays (data not shown). However we found that at low doses of rapamycin (0.1 ng/ml), the NF1 mediated activation of AKT and ERK were unaffected while the NF1 mediated S6-phosphorylation was completely inhibited (Figs. 6A and S2). This dose of rapamycin was also sufficient to completely abrogate the proliferative response observed with the loss of NF1 expression. These data suggest an unexpected obligatory contribution of this pathway to the enhanced proliferation following suppression of NF1 expression. We next investigated the role mTORC1 in regulating two known modulators of endothelial cell cycle progression, Cyclin D1 and the phosphorylation of the corresponding cyclin dependent kinase inhibitor, p27^KIP^ [23]. We find the induction of cyclin D and the phosphorylation of p27 is strongly affected by low dose rapamycin. Collectively these data suggest that the proliferative response we observe in endothelial cells with reduced NF1 expression is highly sensitive to inhibition by rapamycin which seems to act at least in part by suppressing cyclin D induction and the phosphorylation of p27.

**Figure 7:**
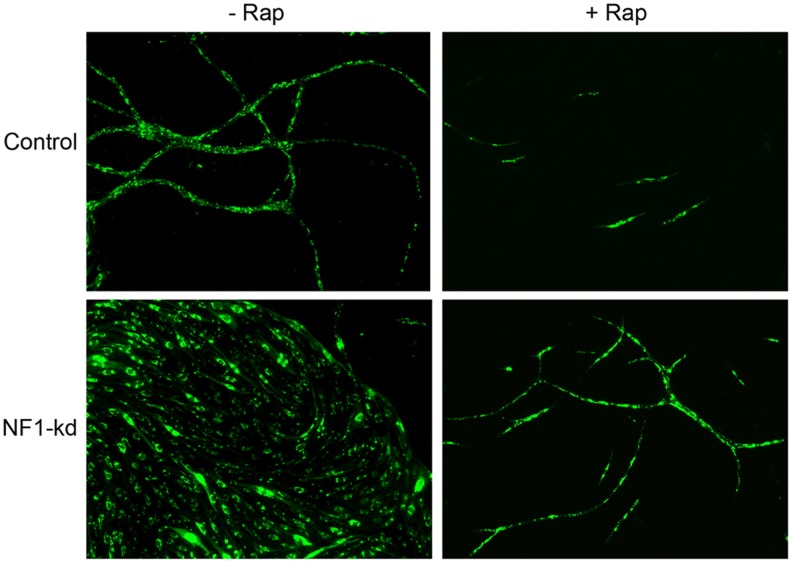
Abnormal vascular morphogenesis is normalized by Rapamycin. Endothelial cells infected with pSIREN expressing either non-targeting (control) or shRNA directed against NF1 (NF1-kd) were plated in co-culture with primary fibroblasts. Co-cultures were incubated in the presence of vehicle (DMSO) or Rapamycin (0.1 ng/ml) from the time of plating. Vascular structures are shown at day 14, visualized by expression of GFP in the endothelial cells by the pSIREN vector.

As cells lacking NF1 induce a hyperplastic phenotype in co-culture, we reasoned that this abnormal morphogenesis might be altered by low dose rapamycin. To test this, control and NF1-kd cells were co-cultured with primary fibroblasts, with or without low doses of rapamycin (Fig. 7). We found that in control endothelial cells the presence of rapamycin inhibited the number of tube-like structures; however the general characteristics of these branching networks were similar to untreated cells. Interestingly, in the NF1-kd cells, the sheet like phenotype was not present, rather the cells underwent normal branching morphogenesis, indicating that the blunting of mTORC1 signaling and endothelial cell proliferation was able to restore a normal phenotype to these cells.

## Discussion

Our data strongly support a critical role for NF1 in the regulation of the vascular endothelium. These findings are in agreement with previous studies done in developmental models whereby the endothelial specific deletion of NF1 resulted in embryonic lethality. These data also agree with the data of Munchhof et al. [8], who found that knocking down of expression in endothelial cells augmented VEGF and FGF signaling and induced angiogenesis. Importantly our data extend this previous work in several critical areas both conceptually and mechanistically.

Notably, our data demonstrate that the loss of NF1 is sufficient to induce activation of Ras and initiate downstream signaling. This suggests that NF1 doesn’t just play a passive role in dampening Ras activation but rather is an active regulator of GTP-Ras accumulation. The loss of NF1 triggers the accumulation of Ras-GTP even under serum and growth factor-free conditions. The resulting accumulation of Ras-GTP is sufficient to initiate changes in cell behavior, including entry into the cell cycle and enhanced growth rates under mitogenic conditions. Importantly, we find no evidence of the senescence associated with the loss of NF1 in other cell types [11]. This finding is consistent with our previous findings showing a lack of senescence following expression of activated H-Ras. The changes in Ras-related signaling result in a gross perturbation of the endothelial cell vasculogenic program. Under co-culture conditions, normal primary endothelial cells stop growing, elongate, and form interconnecting tubules; NF1-kd cells however, continue to maintain at least a partially proliferative phenotype and fail to branch and form tubular structures reliably. To our knowledge this is the first report that the loss of NF1 is sufficient to alter the morphogenic program of endothelial cells. This finding may help to shed light on the underlying cause of a least some of the vascular anomalies seen in some NF1 patients and may provide an *in vitro* model to study the molecular basis for these vascular defects.

It is noteworthy that the alteration of the morphogenic phenotype appears to be quite plastic. Under conditions where the vessel structures are malformed, a return of normal Ras regulation permits at least a partial normalization. Similarly even in stable vessel structures, the loss of NF1 and accompanying Ras activation results in the emergence of an altered phenotype. These data suggest that in NF1 patients, the acquisition of an additional mutation or epigenetic silencing of the remaining copy of NF1 might be sufficient to trigger Ras activation, autonomous proliferation and abnormal vessel formation – even in post-developmental quiescent vasculature. This data begins to explain the high frequency of moyamoya disease, arteriovenous malformations and possibly even aneurysms in the NF1 patient population. The finding that the Ras-GAP, RASA1 (p120-GAP), is mutated in several distinct vascular anomalies [24], [25] including capillary malformation-arterial venous malformations and Vein of Galen aneurysms supports this hypothesis, though it is currently unclear if the loss of RASA1 is sufficient to result in Ras activation. Recent data investigating the role of RASA1 in the endothelium of adult mice suggests that loss of RASA1 does result in abnormal morphology and lymphatic endothelial proliferation. However the activation of Ras and Ras-related signaling, along with the abnormal vascular phenotype were dependent upon coincident VEGFR3 signaling [26]. Current experiments are determining if the homozygous loss of NF1in the post-natal vasculature is sufficient to trigger Ras signaling and vascular anomalies, particularly in a haploinsufficient microenvironment which is known to be pro-angiogenic [7].

Our data directly addressed the role of Ras in the endothelial cell regulation by NF1. While some cell types have been reported to have important Ras-independent functions of NF1 [27], [28], [29], our data suggest that alteration in Ras signaling is essential for the effects seen upon loss of NF1 in endothelial cells. We found that all of the phenotypes and signaling changes observed following the loss of NF1 were rescued by re-expression of the GAP-related domain of NF1 and the restoration of Ras regulation. However it is currently unclear if there is isoform specificity to the Ras activation seen following the loss of NF1 or if particular isoforms are linked to the phenotypic manifestations. Moreover we cannot rule out that other signaling changes are also taking place and contributing to the observed phenotypes.

We find that loss of NF1 triggered several Ras-related signaling pathways including activation of PI-3′-kinase and activation of mTOR signaling. The activation of mTOR was obligatory for the autonomous proliferation triggered by the loss of NF1. The proliferative dependency appears to arise out of mTOR-dependent phosphorylation of p27 leading to its degradation as well as induction of cyclin D1 which has previously been implicated in NF1 tumors [30]. Previous studies in our lab have determined that p27 degradation is an essential step in endothelial progression to S-Phase [23]. Our data do not address whether mTORC1 is directly phosphorylating p27, however we did not find any significant inhibition of either AKT or ERK (two enzymes known to phosphorylate p27) by the low dose of rapamycin we employed. It is interesting to note that activation of mTOR has been associated with cutaneous vascular malformations [31]. The sensitivity of the NF1-kd endothelial cell proliferation and the normalization of the vascular morphogenesis following treatment with low-dose rapamycin in our assays suggest that “Rapalogs” currently in clinical trials as anti-tumor medications may be an effective management tool for certain types of vascular dysfunction in NF1 patients. In addition, it seems likely that these patients may be well-positioned to benefit from other currently approved vasculoprotective therapeutics, e.g., statins which can dampen both Ras and mTOR signaling [32], [33] as well as metformin which activates AMPK signaling [34] to dampen mTOR.

## Materials and Methods

### Ethics Statement

Parents and legal guardians of donors provided consent that discarded tissue could be used for research purposes. The collection and use of tissue samples was evaluated and approved by the Institutional Review Board of Albany Medical Center. It was not considered Human subjects research under 45 CFR (Basic HHS Policy for Protection of Human Research Subjects) part 46 because, (1) the specimens were not collected specifically for the currently proposed research project and (2) the investigator(s) cannot readily ascertain the identity of the individual(s), consistent with the guidelines set forth by the Office of Human Research Protection of the US Federal Government.

### Cell Culture

HUVECs (Human Umbilical Vein Endothelial Cells) were purchased from Cascade Biologics (Portland, Oregon, USA) or Lifeline Cell Technologies (Fredrick MD, USA). Cells were cultured as previously described [21]. Cells were made quiescent by incubation in serum free MCDB-131 supplemented with 1% penicillin/streptomycin and 2 mM L-glutamine (SF), where noted. Stimulation was performed with complete growth media. Primary fibroblasts were isolated from human foreskins provided as de-identified, discarded tissue from neonatal circumcision procedures at Albany Medical Center and grown in DMEM containing 10% FBS and 1% penicillin/streptomycin.

### Western Blotting

Western blotting analysis used the following antibodies: mouse anti-pERK (Santa Cruz Biotechnology), rabbit anti-ERK2 (Santa Cruz Biotechnology), rabbit anti-NF1 (Bethyl Laboratories), rabbit anti-p27 (Santa Cruz Biotechnology), rabbit anti-phospho-p27 (Zymed), rabbit anti-pS6, rabbit anti-pAKT, mouse anti-cyclin D1, mouse anti-pan Ras (Oncogene Research, Calbiochem). All antibodies were used at a dilution of 1∶1000 overnight at 4°C. Other conditions were the same as described in [21] except exposures were captured on a Kodak 4000 MM imager. All figures are representative of at least three independent experiments. Quantification was performed by using volume measurements from the KODAK imager for the band of interest and normalizing to the volume of the loading control (ERK2) for that lane. Data is the mean of three experiments and error bars represent the standard error of the mean. Statistical analysis was performed using students t-test or in some cases ANNOVA with a Bonferroni correction for multivariable conditions.

### Plasmid Construction

To stably knockdown *NF1* expression in HUVECs a shRNA sequence targeting *NF1* (5′gatccGGACACAATGAGATTAGATTTCTCAAGAGAAAATCTAATCTCATTGTGTCCTTTTTTACGCGTg3′ sense strand) and (5′aattcACGCGTAAAAAAGGACACAATGAGATTAGATTTTCTCTTGAGAAATCTAATCTCATTGTGTCCg3′ antisense strand) into the RNAi-Ready pSIREN-RetroQ-ZsGreen retroviral expression vector from Clontech. The shRNA sequences were synthesized by Operon and had a MluI restriction site in the hairpin loop region with BamHI and EcoRI cut overhangs for cloning. The oligonucleotides were annealed and ligated into the BamHI/EcorI cut pSIREN-ZsGreen according to the “Knockout RNAi Systems User Manual” from Clontech. The same annealed shRNAs were ligated into the *BamHI/EcorI* cut pSIREN-RetroQ-DsRed-Express vector to make a knockdown vector expressing red fluorescent protein. A Negative Control shRNA annealed oligonucleotide provided by Clontech was ligated similarly into the pSIREN-RetroQ-ZsGreen to make a control vector. Inducible knockdown of NF1 was achieved using a lentiviral pTRIPZ vector from Open Biosystems carrying the V2THS_260806 sequence for knocking down NF1. Schematics of these vector constructs are shown in *Supplementary Fig. S1*. To make a lentiviral vector expressing the gap related domain (GRD) of NF1 the MSCV-puro-GRD-V5 plasmid was purchased from Addgene. A *BglII/NotI* fragment carrying the GRD was ligated into *BamHI/NotI* cut pEN_TRE2 [10]. The Tet promoter along with NF1-GRD was then put into the Gateway compatible destination vector pSLIK-Venus [35] using an LR-*Clonase*™ reaction resulting in the pSLIK-GRD-Venus plasmid.

### Production and Infection with Retroviruses and Lentiviruses

The pSIREN-RetroQ-ZsGreen lentiviral vector carrying a negative control or a NF1 shRNA was transfected into retroviral packaging PhoenixA cells [36] using Lipofectamine 2000 reagent. The media from the transfected cells was sterile filtered through a 0.4 µM filter and the viral supernatant was used to infect low passage HUVECs in the presence of 5 µg/mL of polybrene. The HUVECs underwent a second round of infection in a similar manner after 24 h. The infection efficiency was 70–80% and a pure population of infected cells was obtained by flow cytometry based sorting under sterile conditions, using ZsGreen as a selectable marker. The lentiviral vectors (2 µg) were co-transfected along with the respective packaging plasmids into 60% confluent 293 FT packaging cells (Invitrogen) cell using Lipofectamine 2000 reagent (Invitrogen). The pSLIK based vectors were co-transfected with three 3^rd^ generation packaging plasmids, 3 µg each of pMDLg/pRRE(Addgene, plasmid 12251), pRSVREV (Addgene, plasmid 12253), the vesicular stomatitis virus (VSV) G envelope plasmid pVSV (Addgene, plasmid 12259) [37]. The pTRIPZ lentiviral vectors were co-transfected along with two 2^nd^ generation packaging plasmids, 3 µg each of pCMV-dR8.2 dvpr (Addgene, plasmid 8455), pCMV-VSVG (Addgene, plasmid 8454). Low passage HUVECs were infected and sorted in a similar manner as above to obtain a pure population of knockdown cells. With both the retroviral and lentiviral vectors, three independent infections were performed on three independent endothelial cell cultures in order to insure representative results.

### Measurement of DNA Synthesis and Growth Assays

Endothelial cells were serum starved for 24 h, after which complete growth media (GM) was added as a mitogenic stimulus for 16 h. Measurements of BrdU incorporation were performed as previously described [22]. In some cases indicated doses of Rapamycin were added to the cells at the time of serum starvation. Growth assays were conducted as described previously [38]. Population doublings were calculated using the formula: Population Doublings = Log(Final cell number/Initial cell number)/Log2. Cumulative population doublings represent the sum of population doublings from all previous passages.

### Co-culture Assay

This assay was performed as previously described [15] with modifications as we have previously reported [10]. Cell were typically tracked using the expression of fluorescent markers introduced during genetic modification. In some cases, cells were stained live with a FITC-tagged UEA-1 lectin (Sigma-Aldrich) or fixed in 3.7% formaldehyde and visualized with UEA-1 lectin.

## Supporting Information

Figure S1 **Schematic representation of NF1 knockdown constructs.**
(TIF)

Figure S2 **Knockdown of NF1 by pSiren activates Ras and cellular signaling.** (A) Cells infected with the pSiren vector targeting a control sequence or NF1 (NF1-kd) were analyzed by western blotting for the expression of NF1 using ERK2 as a loading control *(upper panels)*. Cell lysates were also probed for active Ras using GTP pull-down experiments using anti-Ras immunoblots *(lower panels)*. GTP-Ras represents Ras bound to GST-Raf beads. Levels of total Ras in the input lysate were used to insure similar levels of lysate loading onto the beads. (B) Cell lysates from pSiren infected cells expressing control or NF-kd shRNAs were analyzed for presence of signals known to be downstream of active Ras and for sensitivity to treatment with low doses of the signal transduction inhibitors indicated using immunoblotting. ERK2 is used as a loading control to insure equivalent cellular lysate. Quantification of several similar experiments is shown in Figure 6A.
(TIF)
