# Supplementary figures and images for: Loss of NF1 Expression in Human Endothelial Cells Promotes Autonomous Proliferation and Altered Vascular Morphogenesis
Source: PLoS One. 2012 Nov 7;7(11):e49222. doi: 10.1371/journal.pone.0049222 (PMC3492274; doi:10.1371/journal.pone.0049222)

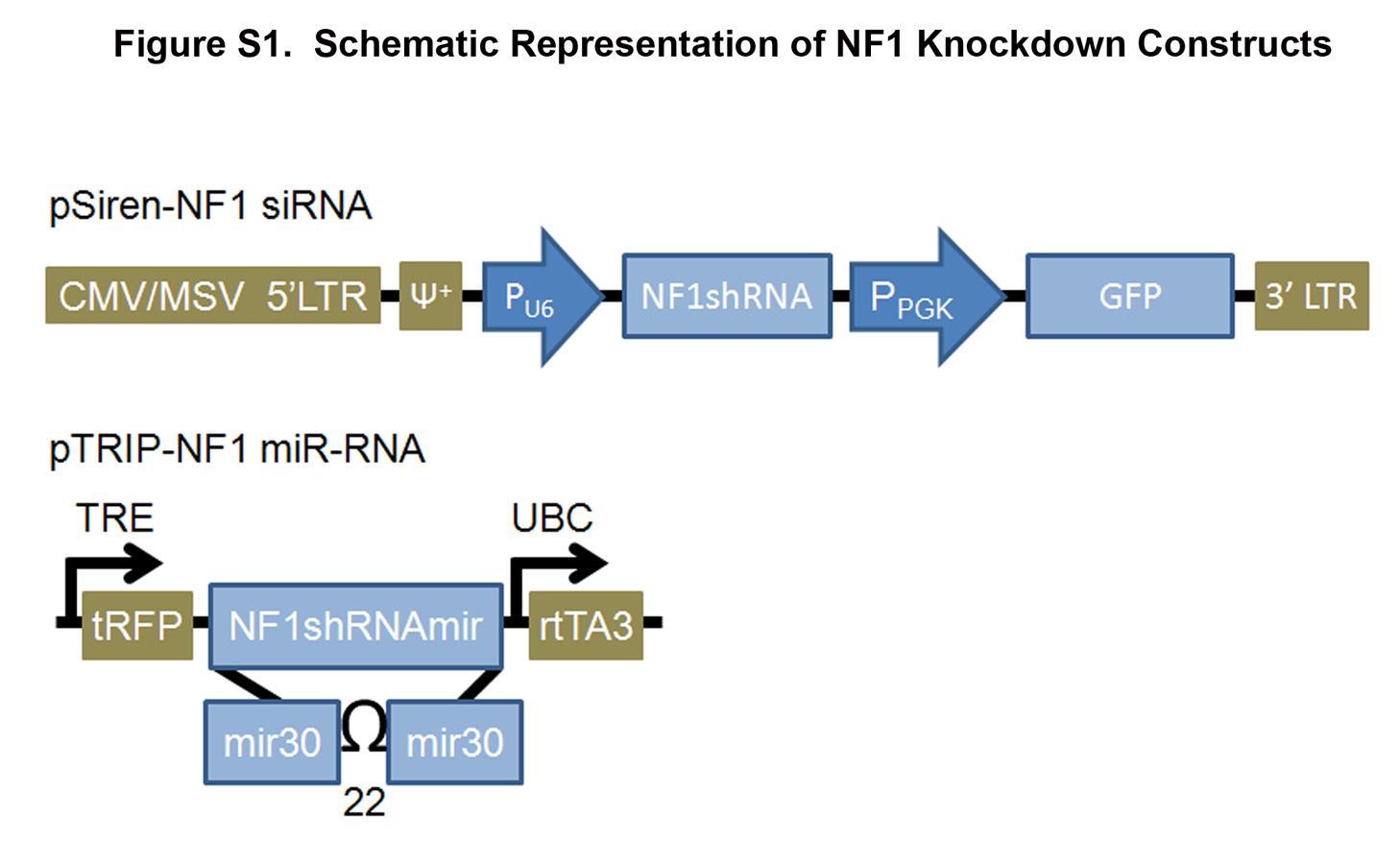

Supplement: Figure S1 — Schematic representation of NF1 knockdown constructs. (TIF) [file pone.0049222.s001.tif]

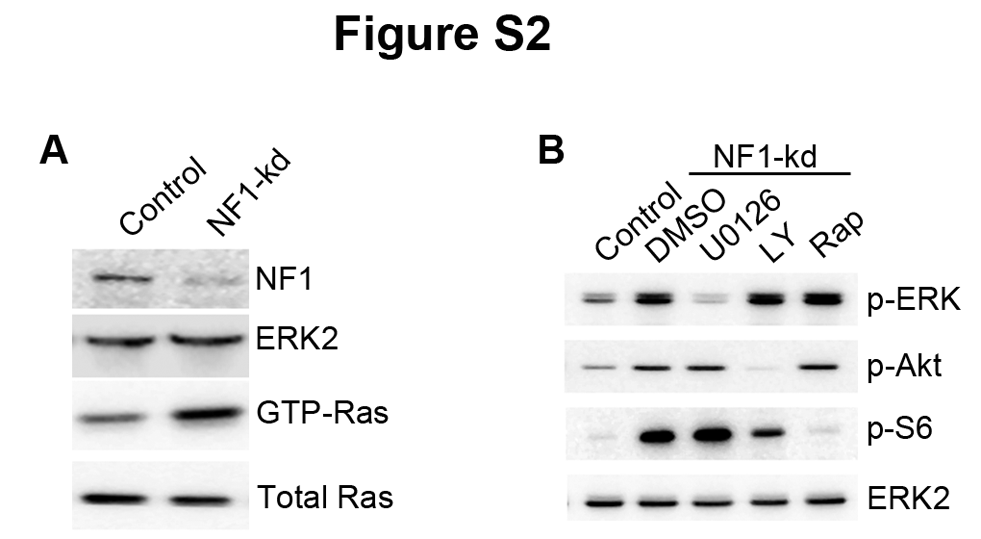

Supplement: Figure S2 — Knockdown of NF1 by pSiren activates Ras and cellular signaling. (A) Cells infected with the pSiren vector targeting a control sequence or NF1 (NF1-kd) were analyzed by western blotting for the expression of NF1 using ERK2 as a loading control (upper panels). Cell lysates were also probed for active Ras using GTP pull-down experiments using anti-Ras immunoblots (lower panels). GTP-Ras represents Ras bound to GST-Raf beads. Levels of total Ras in the input lysate were used to insure similar levels of lysate loading onto the beads. (B) Cell lysates from pSiren infected cells expressing control or NF-kd shRNAs were analyzed for presence of signals known to be downstream of active Ras and for sensitivity to treatment with low doses of the signal transduction inhibitors indicated using immunoblotting. ERK2 is used as a loading control to insure equivalent cellular lysate. Quantification of several similar experiments is shown in Figure 6A. (TIF) [file pone.0049222.s002.tif]
